# Lessons from Climate Reports for the Malaysian Medical Community

**DOI:** 10.21315/mjms2022.29.3.1

**Published:** 2022-06-28

**Authors:** Jemilah Mahmood, Renzo R. Guinto

**Affiliations:** Sunway Centre for Planetary Health, Sunway University, Selangor, Malaysia

## Introduction

The period 2021–2022 is registering a number of alarming reports about the climate crisis. For the past year, the Intergovernmental Panel on Climate Change (IPCC), which is the United Nation’s body responsible for advancing climate science, published the three installments of their Sixth Assessment Report (AR6). These reports, authored by hundreds of scientists, comprised of thousands of pages and citing tens of thousands of research articles, are produced by the IPCC’s three working groups: i) Working Group 1 focusing on the physical science basis of climate change; ii) Working Group 2 examining climate change’s impacts and society’s vulnerability and adaptations to them; and iii) Working Group 3 dealing with current progress in relation to climate mitigation— meaning the reduction of greenhouse gas emissions in order to prevent the worsening of the climate emergency.

## Climate Impacts in Southeast Asia

Compared with the IPCC’s five previous reports issued over the past 30 years, the AR6 is remarkable because of its stronger emphasis on solutions, greater level of integration across sectors and dimensions, and presence of more regional-level information and analysis. The section discussing climate impacts and especially on ecosystems and human health in Southeast Asia, (including Malaysia), is particularly worrisome. Box 1 below summarises highlights these impacts, which are included in the regional brief on Asia prepared by Working Group 2.

Box 1 **Summary of human health and ecological impacts of climate change in Southeast Asia based on the AR6 Working Group 2 Report (1)**

- Rising temperature increases the likelihood of the threat of heatwaves across Asia as well as floods in monsoon regions in South, Southeast and East Asia.
- Observed biodiversity or habitat loss of animals or plants is linked to climate change in some parts of Asia.
- The risk of irreversible loss of coral reefs, tidal marshes, seagrass meadows, plankton community and other marine and coastal ecosystems increases with global warming, especially at 2 °C temperature rise or more.
- Climate change is increasing vector-borne and water-borne diseases, undernutrition, mental disorders and allergic diseases in Asia by increasing hazards such as heatwaves, flooding and drought, air ollutants, in combination with more exposure and vulnerability.
- Increases in heavy rain and temperature will heighten the risk of diarrheal diseases, dengue fever and malaria in tropical and subtropical Asia. More frequent hot days and intense heatwaves will increase heat-related deaths in Asia.
- Due to global warming, Asian countries could experience increase of drought conditions (5%–20%) by the end of this century.
- Increased floods and droughts, together with heat stress, will have an adverse impact on food availability and prices of food resulting in increased undernourishment in South and Southeast Asia.
- In Southeast and East Asia, cyclones, floods and typhoons triggered internal displacement of 9.6 million people in 2019, almost 30% of total global displacements.

## Climate Impacts in Malaysia

The above-mentioned health and ecological impacts in Southeast Asia must be a cause of urgent concern for the Malaysian medical community. Given Malaysia’s geography, many of these regional impacts are highly likely to be felt by Malaysians in the coming years and decades. In fact, the floodings that happened in Kuala Lumpur in late 2021 are a sneak preview of what is yet to come in the future. These regional impacts are reinforced by another report published in 2021, written jointly by the World Bank and the Asian Development Bank, which assessed the climate risk profile of Malaysia. Below are some of the major climatic trends that are anticipated to occur in Malaysia in the future (Box 2).

Box 2 **Summary of expected climatic changes in Malaysia according to the Climate Risk Country Profile published by the World Bank and the Asian Development Bank (2)**

- Between 1970 and 2013, Peninsular Malaysia, Sabah and Sarawak regions experienced surface mean temperature increase of 0.14 °C–0.25 °C per decade. Under a scenario where greenhouse gas emissions are highest, average temperatures are projected to increase by 3.11 °C by the 2090s.
- An increase in rainfall is projected and is expected to be greater in Sabah and Sarawak than in Peninsular Malaysia. Projections for precipitation, while highly variable, shows rainfall likely to increase overall, as well as experience an increase in intensity for extreme rainfall events.
- Malaysia is particularly vulnerable to flooding, with this natural hazard contributing more damage than any other the country experiences. The frequency and extremity of flood events have increased in recent decades with projections showing they will continue to increase with continued global warming.
- The frequency and intensity of heat waves experienced in Malaysia is projected to increase significantly due to a warming climate.
- The sea level rise experienced in coastal areas is expected to reach approximately 0.4 m–0.7 m by 2100, with the magnitude expected to be much greater in Sabah-Sarawak. Coastal inundation resulting from sea level rise poses enormous risks to agricultural production in coastal areas.
- Modelling suggests that occurrence of droughts and floods early in the rice-growing season could reduce yields by up to 60%. Furthermore, drought conditions may impact the cultivation of rubber, palm oil and cocoa.
- In Malaysia, climate change threatens to exacerbate poverty and inequality, with low-income earners, typically living in more exposed areas, are economically dependent on activities where climatic conditions play a prominent role, such as agriculture, fishing and informal sectors in the urban economy.

## Impacts of Climate Change on Malaysian Children’s Health

If climate change is not averted and these local climatic trends continue to manifest, then the health of Malaysians will be negatively impacted. Climate change is no longer just an environmental or economic issue, but a public health crisis that the Malaysian medical community cannot ignore and must give utmost attention to. Climate change impacts all people, but most especially children—those who live today and will become future adults, and those who are yet to be born in the coming decades. And this is where another important report provides evidence. Late in 2021, UNICEF Malaysia collaborated with Universiti Kebangsaan Malaysia and Universiti Malaysia Sabah on producing ‘Impact of climate change on children: A Malaysian perspective’ (3). Informed by case series reviews, desk studies, community cases, analysis of policy and legal documents and engagements with stakeholders, the study revealed worrying interconnections between climate change and environmental degradation and children’s health and well-being in Malaysia.

The UNICEF report is comprehensive, covering the current and projected epidemiology of climate-sensitive diseases affecting Malaysian children, particularly respiratory diseases due to urban air pollution and wildfire haze; existing national policies governing environment, climate change, health, nutrition, and child’s rights in Malaysia as well as the pressing gaps and challenges in implementing them; and the local manifestations of climate impacts on children’s health among Indigenous children in Perak, stateless children in Sabah, and urban poor children in Kuala Lumpur. The report concluded that Malaysia’s governance framework for climate and environment does not adequately consider all aspects of children’s rights and well-being, hence the need for more child-sensitive climate and environmental policies in the country, among others.

## What the Malaysian Medical Community Must Do

These and other reports, provide crystal clear evidence that climate change is already happening in Malaysia, as it is across the world, and is already impacting the health of human populations, especially children. The challenge now is to translate the evidence provided in these reports into action. For knowledge gaps that have been identified by these reports, academic institutions, including medical research institutes, must harness their expertise to find the answers to unknowns at the climate-health interface by conducting more transdisciplinary research. Meanwhile, there is also a need to advocate for national policies that are more ambitious when it comes to tackling the climate crisis, particularly climate mitigation and adaptation. Policies need to be integrative in terms of bringing the health of the planet and the health of people together. The 12th Malaysia Plan, which mentions the term ‘planetary health’ several times, provides a strong foundation, and a good starting point is to revisit the National Policy on Climate Change 2009 and strengthen it further.

Ultimately, to advance the climate and health agenda in Malaysia, there is a need to urgently develop a strong domestic climate and health community. Here the Malaysian medical community must play a leadership role and rally the entire health sector altogether. Public health as an area of specialisation must now be taken more seriously; planetary health needs to be embedded into health policies, and more health professionals need to be exposed to it through training on the wide range of issues it covers. Moreover, because the climate emergency is a local, regional and global challenge, the emerging climate and health community in Malaysia must also forge collaborations and alliances with fellow medical societies, research institutions, and healthcare organizations based in our neighboring countries particularly in member states of the Association of South East Asian Nations (ASEAN) to jointly address head on the shared planetary health challenges discussed in these frightening reports.
